# Relationship between 30 Days Mortality and Incidence of Intraoperative Cardiac Arrest According to the Timing of ECMO

**DOI:** 10.3390/jcm10091977

**Published:** 2021-05-05

**Authors:** Taehwa Kim, Seungeun Lee, Sungkwang Lee

**Affiliations:** 1 Division of Pulmonology, Allergy and Critical Care Medicine, Department of Internal Medicine, Pusan National University Yangsan Hospital, Yangsan 50612, Gyoungnam, Korea; taehwagongju@naver.com; 2 Department of Thoracic and Cardiovascular Surgery, Pusan National University Yangsan Hospital, Yangsan 50612, Gyoungnam, Korea

**Keywords:** extracorporeal membrane oxygenation, thoracic surgery, intraoperative cardiopulmonary resuscitation, intraoperative cardiac arrest

## Abstract

Background: Recently, the use of extracorporeal membrane oxygenation (ECMO) in noncardiac surgery, such as thoracic surgery, has increased. However, there have been no studies on the mortality and incidence of intraoperative cardiac arrest with or without ECMO during thoracic surgery. Methods: Between January 2011 and October 2018, 63 patients received ECMO support during thoracic surgery. All patients who applied ECMO from starting at any time before surgery to the day of surgery were included. Patients were divided into the emergency ECMO group and the non-emergency ECMO group according to the timing of ECMO. We compared the factors related to 30 day mortality using Cox regression analysis. Results: The emergency ECMO and non-emergency ECMO groups comprised 27 and 36 patients, respectively. On the operation day, cardiopulmonary resuscitation (CPR) was a very important result, and only occurred in the emergency ECMO group (*n* = 20, 74.1% vs. 0%, *p* < 0.001). The most common cause of ECMO indication was the CPR in the emergency ECMO group and respiratory failure in the non-emergency ECMO group. There were significant differences in 30 day mortality between the emergency ECMO group and the non-emergency ECMO group (*n* = 12, 44.4% vs. *n* = 3, 8.3%, *p* = 0.001). The Kaplan–Meier analysis curve for 30 day mortality showed that the emergency ECMO group had a significantly higher rate of 30 day mortality than the non-emergency ECMO group (*X*^2^ = 14.7, *p* < 0.001). Conclusions: A lower incidence of intraoperative cardiac arrest occurred in the non-emergency ECMO group than in the emergency ECMO group. Moreover, 30 day mortality was associated with emergency ECMO.

## 1. Introduction

Traditionally, cardiopulmonary bypass (CPB) has been mainly used in thoracic surgery, but recently, using extracorporeal membrane oxygenation (ECMO) has increased in noncardiac surgery, including pulmonary neoplasm, trauma surgery and airway surgery. [1,2] The use of ECMO provides operative stability and can prevent emergencies before surgery. Therefore, ECMO may be an important facilitative therapy for many perioperative emergencies [3]. In particular, some studies have provided sufficient evidence regarding the application of ECMO within lung transplantation, huge mediastinal tumor resection, complicated pulmonary neoplasm, airway surgery, and trauma surgery for operative stability [3,4,5,6,7]. In addition, there is a case that ECMO provided safety and usefulness to patients with unstable hemodynamic and respiratory during massive pulmonary embolism surgery [8]. However, although ECMO has the advantage of being able to provide operative stability, ECMO is still not commonly used in thoracic surgery for CPB.

ECMO can maintain organ perfusion and cardiac pump function and prevent end-organ failure by maintaining systemic blood flow [9]. ECMO functions, such as reducing end-organ damage, can improve patients’ chances of recovery in reversible conditions and prevent developing fatal conditions [3].

Intraoperative cardiac arrest is an unpredictable and unique event, which is directly related to patient outcome and survival rate [10,11]. Intraoperative cardiopulmonary resuscitation (CPR) and its management applied during intraoperative cardiac arrest differ from those applied during the usual cardiac arrest [12].

In a previous study, the average rate of perioperative adverse events in thoracic surgery was 0.2%, excluding those caused by anesthesia, and the most major adverse events related to the operation were cardiac arrest and massive bleeding [13]. ECMO is expected to reduce the adverse events that occur during thoracic surgery in that it can respond urgently in case of cardiac arrest, and in the case of massive bleeding, a catheter capable of massive transfusion is pre-inserted.

However, no studies have focused on the incidence of cardiac arrest with and without ECMO application or the risk factors associated with intraoperative cardiac arrest. Furthermore, it is unclear whether ECMO can reduce the incidence of intraoperative cardiac arrest. Management of intraoperative cardiac arrest is very different across different hospitals and countries [14].

In this study, we investigated the incidence of intraoperative cardiac arrest according to ECMO application in thoracic surgery and studied the risk factors associated with mortality in patients who underwent intraoperative CPR. If an association between applying ECMO and intraoperative cardiac arrest can be demonstrated, this study may provide evidence that ECMO can increase the survival of patients undergoing thoracic surgery, providing a safer surgical environment.

## 2. Material and Methods

### 2.1. Data Collection

We reviewed all patient data to investigate the incidence of intraoperative cardiac arrest according to the timing of EMCO using patient medical records from January 2011 to October 2018. A total of 5804 patients underwent thoracic surgery during the study at Pusan National University Yangsan Hospital, and 63 patients aged >18 years were treated with ECMO. All patients who received ECMO at any time before surgery until the day of surgery were included. The end-point of the surgery day was defined as the time at which the operation was completed. Patients were divided into the emergency ECMO group and the non-emergency ECMO group according to the timing of ECMO, and the clinical outcomes of each group were compared. Emergency ECMO was defined as unplanned insertion, and the non-emergency ECMO was defined as intentional insertion before the operation. In total, 12 patients experienced lung surgery-associated infection, 15 patients required airway surgery, 15 patients had malignancy, and 21 patients required trauma surgery. We collected baseline data on age, sex, body mass index (BMI), acute physiology and chronic health evaluation (APCHE) II score, sequential organ failure assessment (SOFA) score, ASA, intraoperative cardiac arrest, ECMO indication, failed operation, transfusion during operation, ongoing bleeding, OP-related mortality, type of operation, length of hospital stay, ventilator weaning success, ECMO weaning success, and 30 day mortality.

Intraoperative cardiac arrest was defined as any cardiac arrest that occurred during the perioperative and intraoperative periods. All cardiac arrests that occurred after the patient arrived in the operating room, during induction, and at the time of leaving the operating room were treated with extracorporeal cardiopulmonary resuscitation (E-CPR). All cases of intraoperative E-CPR during anesthesia in the operative room were based on anesthesia medical records. This study was approved by the Institutional Review Board (05–2018–071).

### 2.2. ECMO and Anticoagulant Protocol

The ECMO device used was MAQUET PLS^®^ or TERUMO EBS^®^. The ECMO system was composed of an ECMO device, a centrifugal pump, and simplified Bioline-coated circuits. Veno-arterial (VA) ECMO was used for hemodynamic support. Cannulas were inserted via the common femoral artery and femoral veins. Respiratory support was provided via venovenous ECMO. A drainage cannula was inserted via the femoral vein, and the return cannula was inserted via the right internal jugular vein. Before cannula insertion, 50–70 IU/kg heparin was injected intravenously. Generally, additional heparin was not injected because of the risk of bleeding. Activated partial thromboplastin time (ACT) was used to monitor anticoagulation and was maintained between 160 and 190 s. Cannulas were inserted using the Seldinger technique under ultrasound guidance.

### 2.3. Statistical Analysis

Data are expressed as the median and interquartile range (IQR). Descriptive statistics are reported as numbers and percentages. To investigate the clinical outcomes according to the timing of ECMO, baseline characteristics and outcomes were compared between the groups using a *t*-test or Fisher’s exact test, where appropriate. Multivariate Cox regression analyses with a backward stepwise selection method were performed to identify the independent clinical factors of 30 day mortality. Results are expressed as odds ratios (ORs) with 95% confidence intervals (CIs), and two-tailed *p* values of < 0.05 were considered significant. Survival curves and rates were obtained using Kaplan–Meier analyses, and intergroup differences in survival were compared using the log-rank test. Statistical analyses were performed using SPSS version 21 (SPSS, version 21.0; IBM Corp., Armonk, NY, USA).

## 3. Results

### 3.1. Baseline Characteristics of Patients

A total of 63 patients who underwent thoracic surgery with ECMO were divided into the emergency EMCO (*n* = 27) and the non-emergency ECMO (*n* = 36) groups according to the timing of ECMO. Table 1 shows the differences between the two groups (Table 1).

The severity measures for patients, such as APACHE II and ASA, were very higher in the emergency ECMO group than in the non-emergency ECMO group (17.1% vs. 10.8%, *p* = 0.001 and 3.8% vs. 4.3%, *p* < 0.001, respectively).

On the operation day, CPR was a very important result, only occurring in the emergency ECMO group (74.1% vs. 0%, *p* < 0.001). Intraoperative CPR was performed in 19 of 20 CPRs performed (70.4% vs. 0%, *p* < 0.001), indicating that the perioperative or intraoperative CPR rate was very high. This can be considered an unexpected or sudden cardiac arrest. The remaining patient required CPR on the operation day. He was admitted to the emergency room with cardiac arrest and recovered after cardiopulmonary resuscitation.

The rates of emergency operation were also significantly different between the emergency ECMO group and the non-emergency ECMO group (92.6% vs. 33.3%, *p* < 0.001). The most common cause of ECMO indication was CPR in the emergency ECMO group and respiratory failure in the non-emergency ECMO group.

The emergency ECMO group required more transfusions of RBC, FFP, and PLT than the non-emergency ECMO group (22.1 vs. 4.3 packs, *p* < 0.001, 14.7 vs. 2.1 packs, *p* < 0.001, and 10.9 vs. 3.9 packs, *p* = 0.057, respectively).

Estimated blood loss (EBL), postoperative bleeding, ongoing bleeding, and bleeding control were significantly different between the two groups (6205.6 vs. 1188.9 mL, *p* = 0.003; 40.7% vs. 2.8%, *p* < 0.001; 70.4% vs. 19.4%, *p* < 0.001, and 37.0% vs. 2.8%, *p* < 0.001, respectively).

The rate of trauma surgery was higher in the emergency ECMO group than in the non-emergency ECMO group (*n* = 19, 70.4% vs. *n* = 2, 5.6%, *p* < 0.001). However, airway surgery (*n* = 1, 3.7% vs. *n* = 14, 38.9%, *p* = 0.001) and infection surgery (*n* = 1, 3.7% vs. *n* = 11, 30.6%, *p* = 0.007) were more common in the non-emergency ECMO group than in the emergency ECMO group. There were no differences in malignancy surgery between the two groups.

### 3.2. Clinical Outcomes

There were significant differences in 30 day mortality between the emergency ECMO group and the non-emergency ECMO group (*n* = 12, 44.4% vs. *n* = 3, 8.3%, *p* = 0.001) (Table 2). Although there was no difference in the length of postoperative ICU stay and postoperative hospital stay between the two groups, the rates of ventilation weaning (*n* = 13, 48.1% vs. *n* = 27, 75.0%, *p* = 0.028) and ECMO weaning (*n* = 17, 63.0% vs. *n* = 33, 91.7%, *p* = 0.005) were higher in the non-emergency ECMO group than in the emergency ECMO group.

### 3.3. Cox Regression Analysis for Factors Associated with 30 Mortality

Univariate analyses showed OP date CPR (OR: 11.9, 95% CI: 3.1–46.2, *p* < 0.001), intra-OP cardiac arrest (OR: 8.7, 95% CI: 2.4–31.7, *p* = 0.001), APACHE II score (OR: 1.1, 95% CI: 1.0–1.2, *p* = 0.020), ECMO indication (OR: 0.2, 95% CI: 0.1–0.5, *p* < 0.001), OP-related mortality (OR: 188.0, 95% CI: 18.0–1971.8, *p* < 0.001), and ECMO weaning (OR: 0.01, 95% CI: 0.003–0.098, *p* < 0.001) were associated with 30 day mortality (Table 3). All these variables were included in the multivariate analysis, which showed that OP-related mortality (OR: 70.2, 95% CI: 5.5–892.4, *p* = 0.001) and ECMO weaning (OR: 0.1, 95% CI: 0.0–0.7, *p* = 0.021) were associated with 30 day mortality (Table 3).

### 3.4. Proportion of Survivors and Non-Survivors with or without CPR According to the Timing of ECMO

**Figure 1:**
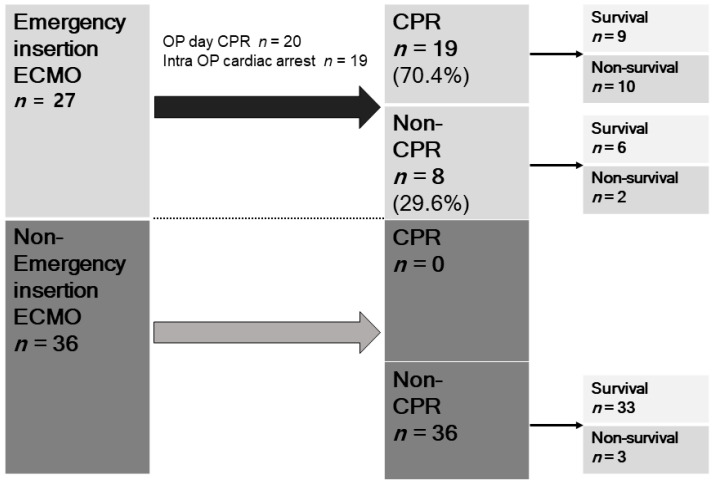
The proportion of survival and non-survival group with or without CPR, in according to the timing of ECMO.

Figure 1 shows the proportion of survivors and non-survivors according to the timing of ECMO. Nineteen patients required intraoperative CPR, and eight patients required non-intraoperative CPR in the emergency ECMO group. Nine of the 19 (47.4%) patients survived in the intraoperative CPR group, while six of eight patients (75.0%) survived in the non-intraoperative CPR group; the 30 day mortality rates were 25.0% and 52.6% (*p* = 0.187), respectively. Non-intraoperative CPR was only required (*n* = 36) in the non-emergency EMCO group. Among patients, who required non-intraoperative CPR, 91.7% (*n* = 33) patients survived, and 8.3% (*n* = 3) patients did not survive.

### 3.5. Kaplan–Meier Analysis for 30-Day Mortality According to Timing of ECMO

**Figure 2:**
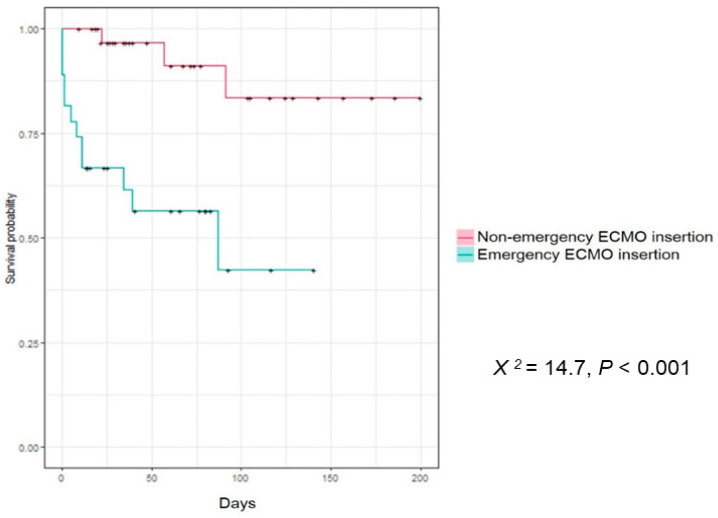
Kaplan–Meier analysis for 30 days mortality according to the timing of ECMO. Kaplan–Meier analysis for 30 days mortality according to the timing of ECMO in emergency EMCO group and non-emergency ECMO group. Emergency ECMO group had a significantly higher 30 days mortality (*X^2^* = 14.7, *p* < 0.001).

The Kaplan–Meier analysis curve for 30 day mortality according to the timing of ECMO in the emergency and non-emergency ECMO groups showed that the emergency ECMO group had a significantly higher 30 day mortality rate than the non-emergency ECMO group (*X*^2^ = 14.7, *p* < 0.001) (Figure 2).

## 4. Discussion

In this study, based on the evidence that ECMO can guarantee the stability of preoperative and perioperative [3,4,5,6,7], it was divided into emergency and non-emergency groups according to the time of application of ECMO based on the time start of operation. If there is a difference in stability according to the time of insertion of the ECMO, adverse events, such as cardiac arrest, will also differ between the two groups. In particular, cardiac arrest is a very important problem and is directly related to patient survival. Therefore, if there is a significant difference between the two groups in the incidence of intraoperative cardiac arrest, the timing of the ECMO insertion could give a very important meaning.

We performed a retrospective analysis of the incidence of intraoperative cardiac arrest, risk factors associated with intraoperative CPR, and 30 day mortality according to the timing of ECMO. To determine whether the incidence of intraoperative cardiac arrest was different according to the timing of ECMO, we compared the emergency and non-emergency ECMO groups.

This study has the following important points. First, we observed that intraoperative cardiac arrest occurred only in the emergency ECMO group (*n* = 19, 70.4%). The most common indication for intraoperative ECMO was CPR (*n* = 14, 51.9%), followed by respiratory failure (*n* = 7, 25.9%) and hemodynamics (*n* = 6, 22.2%). Therefore, the high incidence of intraoperative cardiac arrest in the emergency ECMO group was due to using E-CPR as a treatment for cardiac arrest rather than the characteristics of the group. In other words, some patients had stable vital signs before starting the surgery and underwent surgery without ECMO, but cardiac arrest occurred during preparations for induction (*n* = 5, 26.3%) or during surgery (*n* = 8, 42.1%). In contrast, the intraoperative cardiac arrest did not occur in patients with trauma (*n* = 2, 5.6%) who had unstable initial vital signs and had ECMO inserted before starting the operation. The difference between the two groups was the timing of preoperative or intraoperative ECMO insertion during thoracic surgery. In summary, intraoperative cardiac arrest occurred only during surgery without ECMO, and the incidence of E-CPR was high in the emergency ECMO group. No intraoperative cardiac arrest was observed in the non-emergency ECMO group. This demonstrated that the timing of ECMO was significantly associated with the incidence of intraoperative cardiac arrest during thoracic surgery.

Generally, bleeding is a very important factor in any operation, and a large EBL or transfusion during surgery is a risk factor associated with poor postoperative outcomes and mortality [15,16]. In this study, the most important cause of intraoperative cardiac arrest was massive bleeding during E-CPR. Bleeding refers to all factors related to bleeding, such as ongoing bleeding, transfusion during operation, and EBL. Intraoperative bleeding, which describes EBL and transfusion during surgery, was a significant difference in both groups. In addition, postoperative bleeding, which indicates ongoing bleeding and bleeding control, showed significant *p* values. Previous studies have mainly focused on postoperative outcomes and mortality. However, this study focused on the incidence of intraoperative cardiac arrest associated with risk factors, such as bleeding. A meaningful statistical difference in the incidence of intraoperative cardiac arrest and massive bleeding was identified between the groups, and ECMO cannula was the biggest catheter that could be inserted into the vessels. Thus, through the ECMO circuit, a large amount of blood transfusion and fluid replacement is possible, and it is possible to maintain intravascular volume and cardiopulmonary support [17].

Therefore, using ECMO may prevent intraoperative cardiac arrest in patients with massive bleeding.

Further, there was a significant difference in the 30 day mortality rates between the groups. The reason for this was the difference in the incidence of intraoperative cardiac arrest between the emergency ECMO group and the non-emergency ECMO group. The risk of mortality increases with the increasing incidence of intraoperative cardiac arrest increases [18,19]. This study also confirmed that mortality is correlated with the incidence of intraoperative cardiac arrest. In conclusion, the incidence of intraoperative cardiac arrest differed according to the timing of ECMO, and mortality was high in the group with a high incidence of intraoperative cardiac arrest. Therefore, if it is predicted that hemodynamic instability or respiratory failure may occur during surgery, it is possible to avoid adding ECMO in emergency situations; this would reduce the mortality rate in such situations. Therefore, ECMO may be useful in patients with hemodynamic instability (large mediastinal tumors compressing the heart and great vessels [20], tumors requiring complex pulmonary resection [21], or trauma with massive bleeding [17]) or respiratory failure (large mediastinal mass or pulmonary neoplasm compressing the airways [22], trachea-bronchial surgery with carinal resection [23]) during surgery.

All these cases were unpredictable intraoperative cardiac arrests. This was related to the clinical outcome as 30 days mortality. The stability of the operating room is the most important factor that must be consistently maintained not only for the patients but also for the surgeon and anesthesiologist in charge of the surgery. As a result, surgeons should always watch for intraoperative cardiac arrest and bear in mind that ECMO could be an alternative treatment. This study is very meaningful in that it analyzes the relationship between the timing of ECMO and intraoperative cardiac arrest for the first time.

This study is the first to analyze the relationship between the timing of ECMO and intraoperative cardiac arrest; however, it has several limitations. First, the total number of patients included was small, and the number between groups was also different. Therefore, it is necessary to analyze more cases to provide sufficient information. Second, trauma has a different physiological condition than other diseases. Trauma itself can cause many problems, including massive hemorrhagic complications or derangements in the coagulation system due to consumption or loss of coagulation factors, hypothermia, or acidosis. This is highly related to mortality. However, a closer look at the results of this study showed that trauma did not have an absolute effect on mortality than other diseases. The mortality in the emergency group was 12, among them 8 trauma, 1 airway, and 3 malignant tumors. The mortality in the non-emergency group was 3, and mortality occurred in 1 malignant tumor and 2 infections. This corresponds to 8/21 (38.1%) of the total trauma patients, including the emergency and non-emergency groups, and shows a big difference from the mortality of other disease groups of 7/42 (16.6%), excluding trauma patients. However, reanalyzing only in the emergency situation reflecting that there are no trauma patients in the non-emergency group, trauma mortality was as 8/19 (42%), the other group was 3/8 (37.5%). In this regard, we thought that there might be sufficient bias according to the disease group, but nevertheless, the influence of whether the patient’s condition at the time of ECMO is emergent or non-emergent would be very large, and this study was started. Nevertheless, direct comparison of the trauma group with the other disease group may have influenced the interpretation of the results. Finally, there is a limitation that levels of management and treatment for ECMO are all different.

Generally, the ECMO insertion time is longer than the insertion time of the central line. Therefore, in a group without enough experience, attempts to insert ECMO in urgent situations may be more dangerous. This is why not all hospitals may have the same results as this study.

## 5. Conclusions

In conclusion, we demonstrated that the lower incidence of intraoperative cardiac arrest was in the non-emergency ECMO group based on the timing of ECMO. Moreover, 30 days mortality was significantly associated with the timing of ECMO. It was very high in the emergency ECMO group. Our study suggests that ECMO may have benefits for a safe surgical environment and patient safety.

## Figures and Tables

**Table 1:** Basal characteristics of patients with emergency and non-emergency ECMO groups.

Variable		Timing of ECMO		
	Total *n* = 63	Emergency *n* = 27	Non-Emergency *n* = 36	*p*-Values
Age, years (median, IQR)	54 (40–59)	50 (39–59)	54.5 (40.25–59.75)	0.880
Sex (male)	42 (66.7)	20 (74.1)	22 (61.1)	0.280
BMI, kg/m^2^ (median, IQR)	22.8 (19.8–25.4)	23.3 (20.5–25.8)	21.36 (19.05–25.16)	0.609
APACHE II (median, IQR)	12 (9–16)	16 (12–19)	11 (7–14)	0.001
SOFA (median, IQR)	10 (6–13)	11 (7–13)	10 (4–13.75)	0.209
ASA (median, IQR)	4 (3–5)	5 (4–5)	3 (3–4)	<0.001
Inotropics † (*n*)	33 (52.4)	22 (81.5)	11 (30.6)	<0.001
Emergency OP (*n*)	37 (58.7)	25 (92.6)	12 (33.3)	<0.001
OP day CPR (*n*)	20 (31.7)	20 (74.1)	0 (0)	<0.001
Intra OP cardiac arrest (*n*)	19 (30.2)	19 (70.4)	0 (0)	<0.001
ECMO indication				
CPR (*n*)	14 (22.2)	14 (51.9)	0 (0)	<0.001
Hemodynamic (*n*)	7 (11.1)	6 (22.2)	1 (2.8)	<0.001
Respiratory (*n*)	42 (66.7)	7 (25.9)	35 (97.2)	<0.001
Failed OP ‡ (*n*)	7 (11.1)	7 (25.9)	0 (0)	0.001
OP time, min (median, IQR)	250 (140–340)	180 (135–290)	282.5 (160–343.75)	0.135
Transfusion during OP				
RBC, pack (median, IQR)	3 (0–20)	16 (2–44)	0 (0–3)	<0.001
FFP, pack (median, IQR)	1 (0–11)	9 (0–30)	0 (0–2.75)	<0.001
PLT, pack (median, IQR)	0 (0–12)	0 (0–16)	0 (0–0)	0.057
EBL, mL (median, IQR)	1000 (300–3500)	2500 (1000–10,000)	500 (200–1175)	0.003
Post OP bleeding + (*n*)	12 (19.0)	11 (40.7)	1 (2.8)	<0.001
Bleeding control (*n*)	11 (17.5)	10 (37.0)	1 (2.8)	<0.001
Ongoing bleeding * (*n*)	26 (41.3)	19 (70.4)	7 (19.4)	<0.001
OP related mortality (*n*)	13 (20.6)	10 (37.0)	3 (8.3)	0.005
OP Type				
Trauma (*n*)	21 (33.3)	19 (70.4)	2 (5.6)	<0.001
Airway (*n*)	15 (23.8)	1 (3.7)	14 (38.9)	0.001
Infection (*n*)	12 (19.0)	1 (3.7)	11 (30.6)	0.007
Malignancy (*n*)	15 (23.8)	6 (22.2)	9 (25.0)	0.798

ECMO: extracorporeal membrane oxygenation, BMI: body mass index, APACHE II: acute physiologic assessment and chronic health evaluation II, SOFA: sequential organ failure assessment, ASA: American Society of Anesthesiologists, OP: operation, CPR: cardiopulmonary resuscitation, ECPR: extracorporeal cardiopulmonary resuscitation, RBC: red blood cell, FFP: fresh frozen plasma, PLT: platelet, EBL: estimated blood loss. † Inotropics is defined as an application of any one of vasopressin, norepinephrine, dopamine, dobutamine and epinephrine. ‡ Failed OP was defined as the death of the patient. * Ongoing bleeding was a case where the bleeding continues even after the surgery but stopped due to medical treatment, such as transfusion and hemostatics. + Postop bleeding was when there was much bleeding that requires surgical control. The criteria for the amount of bleeding were set at 100 cc per hour in our center.

**Table 2:** Primary clinical outcome.

		Timing of ECMO		
Variable	Total *n* = 63	Emergency *n* = 27	Non-Emergency *n* = 36	*p*-Values
30 days mortality (*n*)	15 (23.8)	12 (44.4)	3 (8.3)	0.001
Post OP ICU stay (day)	6 (2–28)	5 (1–26)	6.5 (3–33.25)	0.479
Post OP hospital stay (day)	23 (12–69)	14 (1–77)	34 (19–65.75)	0.146
Ventilator weaning (*n*)	40 (63.5)	13 (48.1)	27 (75.0)	0.028
ECMO weaning (*n*)	50 (79.4)	17 (63.0)	33 (91.7)	0.005

ECMO: extracorporeal membrane oxygenation, ICU: intensive care unit, OP: operation.

**Table 3:** Cox regression analysis for factors associated with 30 mortality.

	Univariate Analysis		Multivariate Analysis	
	OR (95% CI)	*p*	OR (95% CI)	*p*
OP date CPR	11.9 (3.1–46.2)	<0.001		
Intra OP cardiac arrest	8.7 (2.4–31.7)	0.001		
APACHE II	1.1 (1.0–1.2)	0.0020		
ECMO indication	0.2 (0.1–0.5)	<0.001		
OP related mortality	188.0 (18.0–1971.8)	<0.001	70.2 (5.5–892.4)	0.001
ECMO weaning	0.01 (0.003–0.098)	<0.001	0.1 (0.0–0.7)	0.021

OR: odds ratio, OP: operation, CPR: cardiopulmonary resuscitation, APACHE II: acute physiologic assessment and chronic health evaluation II, ECMO: extracorporeal membrane oxygenation.

## Data Availability

The datasets used and/or analyzed during the current study are available from the corresponding author on reasonable request.

## Abbreviations

ECMO: extracorporeal membrane oxygenation, CPB: cardiopulmonary bypass, CPR: cardiopulmonary resuscitation, BMI: body mass index, APCHE II score: acute physiology and chronic health evaluation II score, SOFA: sequential organ failure assessment, ASA: anesthesiologist physical status, E-CPR: extracorporeal cardiopulmonary, EBL: estimated blood loss.
